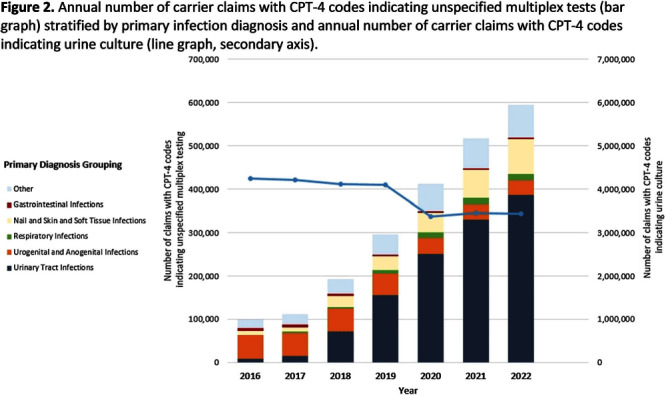# Utilization of multiplex molecular panels for urinary tract infections, Medicare claims, 2016 – 2022

**DOI:** 10.1017/ash.2024.210

**Published:** 2024-09-16

**Authors:** Kelly Hatfield, Sarah Kabbani, Dustin Currie, Christine Kim, Isaac See, Kara Jacobs Slifka, Shelley Magill, Lauri Hicks, Lawrence McDonald, John Jernigan, Sujan Reddy, Joseph Lutgring

**Affiliations:** Centers for Disease Control and Prevention

## Abstract

**Background:** Multiplex molecular tests for infectious diseases can provide highly sensitive results rapidly; however, these tests may more readily detect asymptomatic colonization. There are reports of non-FDA approved laboratory-developed multiplex tests for the diagnosis of urinary tract infections (UTI). Differentiating UTI from asymptomatic bacteriuria is challenging, especially in older adults. The increased sensitivity of multiplex tests may exacerbate this challenge. We sought to describe the use of multiplex testing for UTIs in Medicare claims. **Methods:** Multiplex testing was identified using carrier claims submitted by non-institutional providers using the Chronic Conditions Warehouse for 2016 – 2022. Because there are no CPT-4 codes specifying UTI multiplex testing, we included claims as described in Figure 1 and categorized claims based on the primary ICD-10-CM diagnosis. The payment amounts for line items related to testing for infectious agents were summed. Laboratories were counted using CLIA numbers listed on corresponding claims. Beneficiaries residing in a nursing home at the time of their claim were identified using stay information derived from the Minimum Dataset 3.0. For comparison, similar characteristics among carrier claims with a CPT-4 code indicating urine culture were also described. **Results:** Claims for unspecified multiplex molecular tests overall have increased, driven by increases in claims with a primary UTI diagnosis (from 8,521 in 2016 to 386,943 in 2022), while urine cultures have not (Figure 1). In 2022, 65% of all unspecified multiplex tests were linked to a diagnosis of UTI; UTI multiplex claims were associated with 647 laboratories. For UTI claims, the median cost per claim for line items related to multiplex testing was $589 compared to $13 for urine culture-related line items. Overall, 8% of UTI multiplex claims were for beneficiaries residing in a nursing home. **Conclusions:** Claims for non-FDA approved unspecified multiplex tests associated with a primary diagnosis of UTI have increased >45-times between 2016-2021 and have >45-times higher median costs than urine cultures. The use of this testing in the Medicare population, including nursing home residents, is of potential concern given that inappropriate treatment of asymptomatic bacteriuria has been described to be common in older adults. Research is needed to outline use cases where UTI multiplex testing may be beneficial. Appropriate use of diagnostic testing is important to minimize diagnostic errors and avoid unnecessary antibiotic use.

**Figure:**
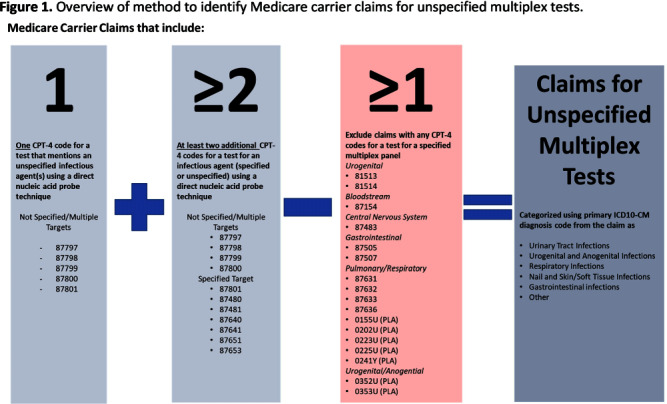

**Figure:**